# Microorganisms in the rumen and intestine of camels have the ability to degrade 2‐amino‐3‐methylimidazo[4, 5‐f]quinoline

**DOI:** 10.1002/fsn3.4115

**Published:** 2024-03-18

**Authors:** Jialing Lin, Chuanhui Zeng, Xueli Li, Qin Tang, Jing Liao, Yan Jiang, Xianchun Zeng

**Affiliations:** ^1^ Chengdu Medical College School of Laboratory Medicine Chengdu Sichuan China; ^2^ Solid‐State Fermentation Resource Utilization Key Laboratory of Sichuan Province Yibin Sichuan China; ^3^ Sichuan Tianfu New District People's Hospital Chengdu China; ^4^ Xinjiang Urumqi Traditional Chinese Medicine Hospital Urumqi Xinjiang China; ^5^ Meat Processing Key Laboratory of Sichuan Province Chengdu Sichuan China

**Keywords:** camel digestive tract, heterocyclic amines, microbial degradation, quinoline

## Abstract

Heterocyclic amines (HAs) are a group of mutagenic and carcinogenic compounds produced from the processing of high‐protein foods, which include 2‐amino‐3‐methylimidazo[4, 5‐f]quinoline (IQ) showing the strongest carcinogenic effect. Camels are able to digest HAs in foods, which provide rich microbial resources for the study. Thus, camel rumen and intestinal microbiota were used to degrade IQ, and the dominant microorganisms and their degradation characteristics were investigated. After three generations of culture with IQ as the sole carbon source, the highest abundance in rumen and intestinal microbes was found in the Proteobacteria phylum. The strains of third generation of the rumen contents were mainly attributed to the genera *Brevundimonas* and *Pseudomonas*, and the dominant genera in intestine were *Ochrobactrum*, *Bacillus*, and *Pseudomonas*. Microorganisms were further isolated and purified from the third generation cultures. These 27 strains from the rumen (L1–L27) and 23 strains from the intestine (C1–C23) were obtained. Among them, four strains with the most effective degrading abilities were as follows: L6 (28.55% of IQ degrading rate) and C1 (25.19%) belonged to the genus *Ochrobactrum*, L15 (23.41%) belonged to the genus *Pseudomonas*, and C16 (20.89%) were of the genus *Bacillus*. This study suggested the application of abundant microbial resources from camels' digestive tract to biodegrade foodborne toxins.

## Graphical abstract

This work was the first to obtain IQ‐degrading bacteria from digestive tract of camels and to reveal the characteristics of these degrading bacteria. Results provided microbial resources for biodegradation of foodborne toxins.

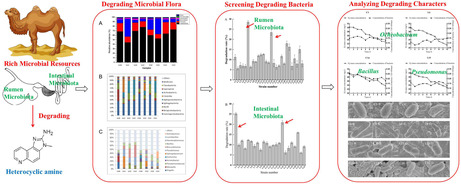

## 1 INTRODUCTION

Heterocyclic amines (HAs) with a polycyclic aromatic structure can cause pancreatic, gastric, and liver cancers (Tanaka et al., 1985). However, more than 30 kinds of HAs have been detected in a variety of fried foods and cured or marinated meat products (Fei et al., 2007). Among these heterocyclic compounds, 2‐amino‐3‐methylimidazo[4,5‐f]quinoline (IQ) is the strongest carcinogenic and has been classified by the International Agency for Research on Cancer as a highly suspicious carcinogen for humans (Class 2A) (Lai et al., 2022). Bylsma found that excessive intake of IQ increases the risk of breast, colon, and stomach cancer in humans (Bylsma & Alexander, 2015). Therefore, the prevention and removal of HAs is of great importance to food safety and human health.

In recent years, it has been found that animal intestinal flora had a certain degradation effect on toxic and harmful substances (Shao et al., 2022; Zeng et al., 2022). HAs could be biodegraded by gut microbiota, but the mechanism needs to be further investigated (Shao et al., 2022).

Ruminant digestive tract microorganisms have evolved into a complex microbial ecosystem, which can degrade phycotoxins, cyanobacterial toxins, and mycotoxins (Loh et al., 2020). Camels are characteristic ruminant animals in Xinjiang, China, often kept in a semiwild state and have biological characteristics such as tolerance to roughage, drought, heat, cold, and salinity. Early studies found that camels have the ability to tolerate or degrade phytotoxins such as mimosine and *Euphorbia esula* toxins (Goel et al., 2005; Kronberg et al., 2006). Toxic plants are often found in the desert where camels live, including *Tamarix chinensis*, *Alhagi sparsifolia*, *Peganum harmala*, and *Stellera chamaejasme* (An et al., 2010). These plants contain phytotoxins, such as seeds of *Peganum harmala*, which are rich in harmine and can elevate body temperature and cause neurological disorders. The safe consumption of these toxic plants by camels is closely related to the microorganisms in their digestive tracts (Gharechahi et al., 2015). Camel rumen microbes have been shown to tolerate and degrade harmine (An et al., 2010), which has a structure similar to HAs, so it is speculated that the microbes in their digestive tract may have the ability to degrade HAs.

Therefore, camel rumen and intestinal contents which contain a variety of microorganisms were investigated to degrade IQ. The microbial diversity including Chao1 index, Shannon index, Goods coverage, and relative abundance was studied. Dominate microbes in the two contents were screened and their degradation capacity was also determined. Results provided microbial resources for biodegradation of foodborne toxins.

## 2 MATERIALS AND METHODS

### 2.1 Sample collection

The experimental camels from the Tianying slaughterhouse in Xinjiang were all healthy before sacrifice. They were fed with grass in the wild. And fodder was provided for camels in the winter when grass was hard to grow. Thus, these animals lived in a semiwild state. Feces from three male camels were collected as intestine samples. Meanwhile, rumen fluids from these camels were kept as rumen contents. Samples were conserved in sterile and anaerobic bags (Shanghai Laichuang Biotechnology Co., LTD) before back to the laboratory and stored at −80°C in the laboratory.

### 2.2 Culture media and reagents

Inorganic salt medium: 2.65 g of KH_2_PO_4_, 4.26 g of Na_2_HPO_4_, 0.01 g of FeSO_4_·7H_2_O, 0.2 g of MgSO_4_·7H_2_O, 0.002 g of MnSO_4_·7H_2_O, 0.02 g of CaCl_2_, 1 g of NH_4_NO_3_, 1000‐mL distilled water, pH 7.0, sterilized at 121°C for 20 min.

LB liquid medium: 5‐g yeast leaching powder, 10‐g sodium chloride, 10‐g peptone, 1000‐mL distilled water, pH 7.0, sterilized at 121°C for 20 min.

Solid isolation medium (g/L): Inorganic salt medium with 15–20 g of agar powder.

IQ standards (Aladdin, chromatographically pure), SuPrep gel DNA extraction kit, OMEGA soil DNA extraction kit, upstream primer 27F (5′‐AGAGTTTGATCATGGCTCAG‐3′), and downstream primer 1492R (5′‐TACGGTTACCTTGTTACGACTT‐3′) were purchased from Shanghai Biotechnology Co., LTD.

### 2.3 Microbial community structure of the camel's digestive tract in response to IQ

Five milliliters of the rumen liquid or 5 mg of intestinal contents from camels was inoculated in 100‐mL inorganic salt medium with 30 mg/L IQ as the sole carbon source in anaerobic bags at 37°C for 10 days, which was regarded as the first generation. Five milliliters of the mixture was then moved into fresh medium with the same culture conditions for another 10 days, which was regarded as the second generation. Then, 5 mL of the mixture was incubated in fresh medium for the third 10 days, as the third generation. Thus, camel rumen contents were cultured with IQ stressed for 0, 10, 20, and 30 days, respectively, named as LG0, LG1, LG2, and LG3. Intestinal contents for 0, 10, 20, and 30 days were named as CG0, CG1, CG2, and CG3, respectively. Replacing samples by equal milliliters of sterile inorganic salt medium in the same culture conditions was set as blank groups.

Total DNA was extracted from every generation (three replicates) using the OMEGA Soil DNA Extraction Kit. DNA mass and concentration were determined using a nanoDrop 2000 assay (A260/280 and A260/230) and stored at −20°C for backup.

PCR amplification was performed using the bacterial universal primers 16S rRNA V4 high variant region 515F (5′‐GTGCCAGCMGCCGCGGTAA‐3′) and 909R (5′‐CCCCGYCAATTCMTTTRAGT‐3′) as primers (Li et al., 2014). The reaction system was 25 mL of total volume with 1.5 mmol/L MgCl_2_, 0.4 mol/L deoxynucleoside triphosphate, 1.0 μmol/L primers, 0.5 U Ex Taq, and 10 ng of total genomic DNA. Reaction procedure: 94°C for 3 min predenaturation; 94°C for 40 s; 56°C for 60 s; 72°C for 60 s; 30 cycles, 72°C extension for 10 min. Meanwhile, sterile deionized water was used in control groups.

QIIME Pipeline‐Version 1.7.0 software was used for processing, and preprocessing to remove low‐quality raw sequencing data before removing chimeric sequences using UChime (Edgar et al., 2011). The operational taxonomic unit (OTU) was based on 97% sequence similarity for each sample for Goods coverage, Chao1, and Shannon diversity indices. The QIIME platform was also used to analyze the differences in microbial community structure among these three generations of samples using principal coordinate analysis (PCoA). The relative abundance of IQ‐degrading bacteria in the camel's digestive tract at the phylum, class, and genus levels were mapped.

### 2.4 Isolation, purification, and identification of IQ‐degrading bacteria

The third generation of camel rumen or intestinal contents was incubated with IQ of 30 mg/L in solid isolation medium at 37°C for 10 days inside anaerobic bags. Then, single colonies as degrading bacteria in the mixture were isolated and purified.

The DNA of these degrading bacteria was extracted using OMEGA soil DNA extraction kit and amplified using 16S rRNA gene sequences with 27F (5′‐AGAGTTTGATCCTGGCTCAG‐3′) and 1492R (5′‐GGTTACCTTGTTACGACTT3′) as primers. The obtained PCR products were sent to Shanghai Biotech Company for sequencing. The neighbor‐joining method was applied to the MEGA6 software to analyze the sequence alignment system, phylogenetic trees were constructed and homologies between strains were calculated.

### 2.5 IQ degradation rate

The degraded strain was incubated in LB medium at 37°C for 24 h and the supernatant was collected by centrifugation. The organisms were washed three times with inorganic salt medium to produce a suspension with 2.0 optical density at 600 nm. Five milliliters of the suspension was inoculated into 95‐mL inorganic salt medium containing 30 mg/L of IQ as the only carbon source at 37°C in anaerobic bags. The suspension was replaced by equal volume of sterile inorganic salt medium as blank control. Samples were taken at 24‐h intervals and incubated for 10 days. Dichloromethane (chromatographically pure) was applied to samples, and the IQ was extracted by centrifugation after ultrasonic shaking for 20 min.

IQ contents were measured by high‐performance liquid chromatography with Agilent Eclipse XDB‐C18 column (250 mm × 4.6 mm × 5 μm). Mobile phase as methanol (chromatographic purity), 1.0 mL/min of flow rate, column temperature was set at 25°C, detection wavelength as 260 nm, quantification ring injection volume as 20.00 μL. The standard equation for IQ was *y* = 192.97*x* + 4.0772, *R* ^2^ = .9994, with the range of 0~40 mg/L IQ.

The IQ concentration of blank control was recorded as A, and the residual concentration of IQ after incubating with bacteria from camel's digestive tract was B; thus, the IQ degradation rate was calculated as (A–B)*100%/A.

### 2.6 Degradation characteristics of IQ‐degrading bacteria

The efficient degradation strain was inoculated with IQ concentration of 30 mg/L in inorganic salt medium at 37°C for 10 days. The blank control was IQ in inorganic salt medium without the addition of bacteria. Samples were taken every 24 h. The bacterial concentration of the culture medium was measured at 600 nm using an ultraviolet spectrophotometer, and the growth curve of the strain was plotted. Meanwhile, the residual amount of IQ in the culture solution was determined by high‐performance liquid chromatography, and the degradation curve was plotted. Two milliliters of the bacterial solution was taken and collected, and then 4% formaldehyde was added for fixation. A small amount of the bacterial solution was picked and placed on a copper grid, dried, and observed by scanning electron microscopy.

### 2.7 Statistical analysis

Statistical analysis was performed with the software Origin 2021Pro (OriginLab, Northampton, MA). All experiments were conducted in triplicate. Data were expressed as average values.

## 3 RESULTS

### 3.1 Evaluation of sequencing quality

Basic information on the original and three‐generation samples of camel rumen or intestine is shown in Table 1. A total of 154,129 sequences were obtained by high‐throughput sequencing of the eight experimental samples, with basic sequence numbers above 10,576 and sequence lengths of about 410 bp.

**TABLE 1:** Basic information on high‐throughput sequencing samples.

Sample	Sequence	Bases (bp)	Sample	Sequence	Bases (bp)
LG0	19,647	8,055,270	CG0	14,816	6,074,560
LG1	15,455	6,336,550	CG1	21,592	8,852,720
LG2	25,773	10,566,930	CG2	24,282	9,955,620
LG3	24,356	9,985,960	CG3	21,208	8,695,280

*Note*: LG0: Original camel rumen sample; LG1, LG2, and LG3: Camel rumen microorganisms from the first‐ to third‐generation samples stressed with IQ; CG0: Original camel intestinal sample; CG1, CG2, and CG3: Camel intestinal microorganisms from the first‐ to third‐generation samples stressed with IQ. The same is below.

**FIGURE 1:**
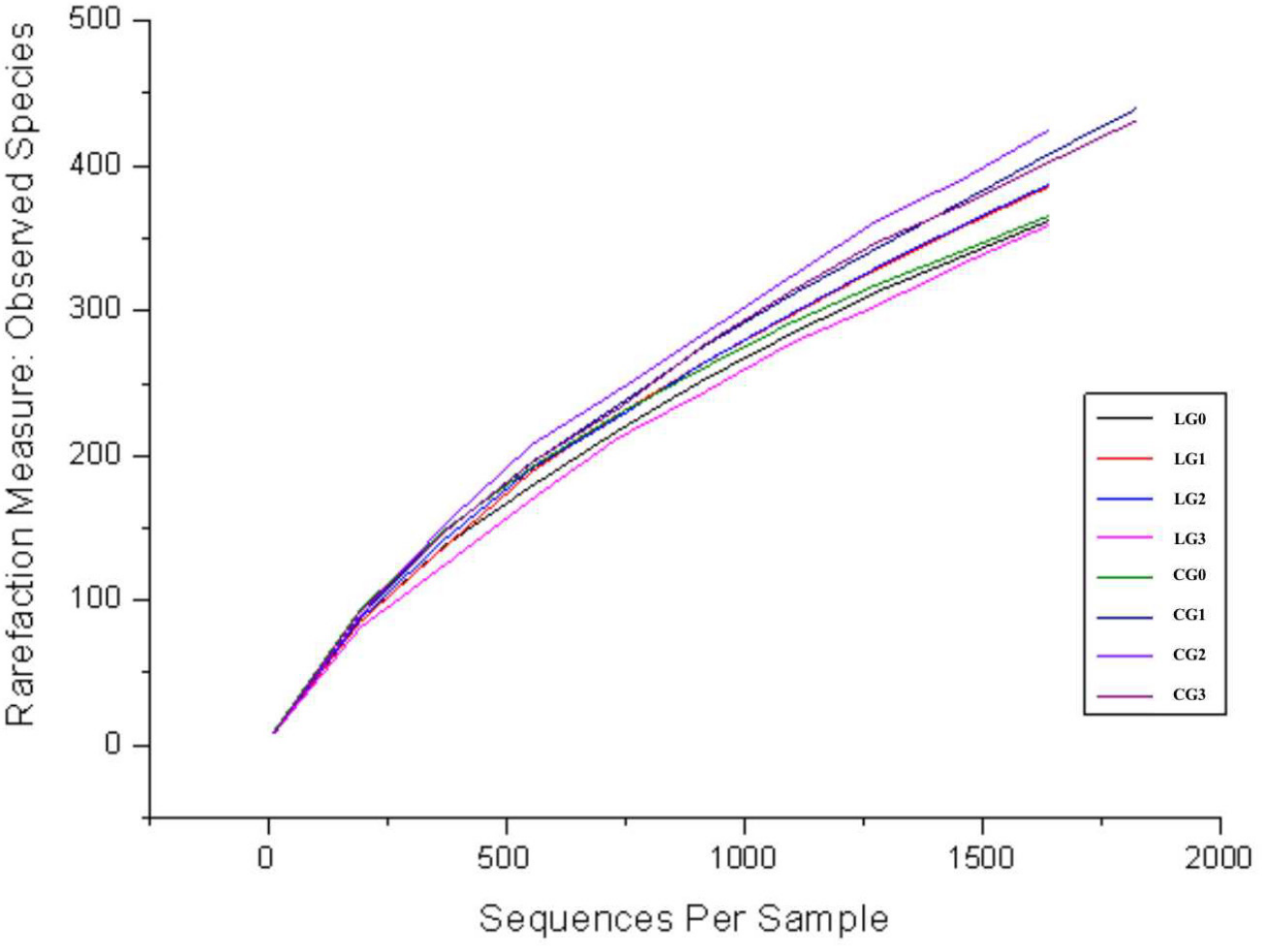
Rarefaction curve of rumen and intestinal samples from camels.

All samples were plotted at 97% similarity level with rarefaction curves as shown in Figure 1. As seen in Figure 1, each sample showed a similar trend of variation. OTUs of all samples were increased. The increasing trend of OTUs of every sample gradually became slower as the number of tests increased, and basically saturated in the final. The sampling depth as shown in Figure 1 and the amount of sequencing data in Table 1 suggested that the sequencing quality of the bacterial flora from the digestive tract of camels with IQ stress was reasonable.

### 3.2 Analysis of microbial diversity in the digestive tract of camels

The alpha diversity of LG0–LG3 and CG0–CG3 is shown in Table 2: Goods coverage ranged between 82.6% and 87.2%, indicating that most information on the bacterial species in these samples was captured. Chao1 index and observed OTU represented species richness, which showed higher values in CG0‐CG3 than LG0‐LG3. Thus, species of microorganisms in the intestinal tract were richer than in the rumen of camels. Shannon's and Simpson's indices presented species diversity and evenness. And high values of the index meant high diversity and abundance of IQ‐degrading bacteria in the intestine as well as rumen of camels.

**TABLE 2:** Microbial diversity indices of samples.

Sample	Chao1	Observed	Shannon	Simpson	Goods coverage (%)
		OTU	Index	Index	
LG0	869.86	405.6	6.47	0.956	87.2
LG1	863.77	386.2	6.45	0.958	85.2
LG2	915.12	387.7	6.29	0.949	84.8
LG3	915.22	359.3	6.00	0.940	85.7
CG0	928.95	398.5	6.98	0.976	86.0
CG1	1185.05	408.5	6.59	0.965	83.9
CG2	1181.83	425.2	6.51	0.938	82.6
CG3	1014.56	402.9	6.68	0.966	84.7

**FIGURE 2:**
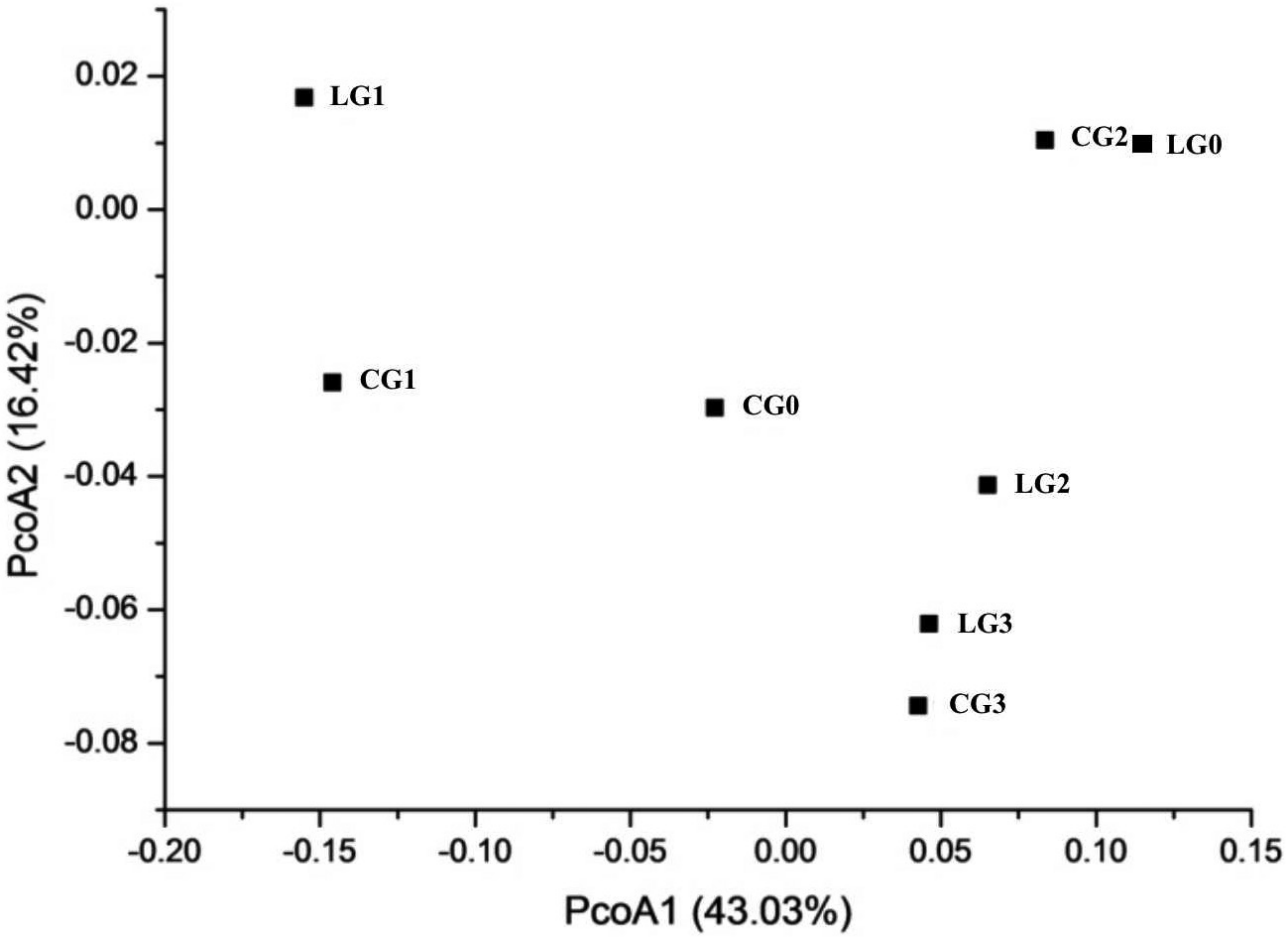
Principal coordinate analysis of rumen and intestinal samples from camels.

Beta diversity of samples was calculated by Weighted UniFrac PCoA analysis to reflect the differences in bacterial communities of these samples (Figure 2). Those clustered together indicated little difference, and those far apart indicated a comparatively large difference. The results showed that there were relative differences in community structure among the original samples of camel rumen (LG0) and intestine (CG0) with the samples cultured under IQ stress (LG1, LG2, LG3, CG1, CG2, and CG3). The IQ‐degrading microbe flora in the rumen of camels differed between every generation (LG1, LG2, and LG3). The microbial taxa that degraded IQ in the camel intestine also showed large differences in all generations (CG1, CG2, and CG3). Interestingly, bacterial communities from the third generation of rumen and intestine contents might be similar, as the two samples (LG3 and CG3) clustered. Clustered LG3 and CG3 likely resulted from incubating three generations with IQ as the only carbon source. Therefore, the bacterial composition in the digestive tract of camels was further investigated.

### 3.3 Community composition of IQ‐degrading bacteria in the digestive tract of camels

**FIGURE 3:**
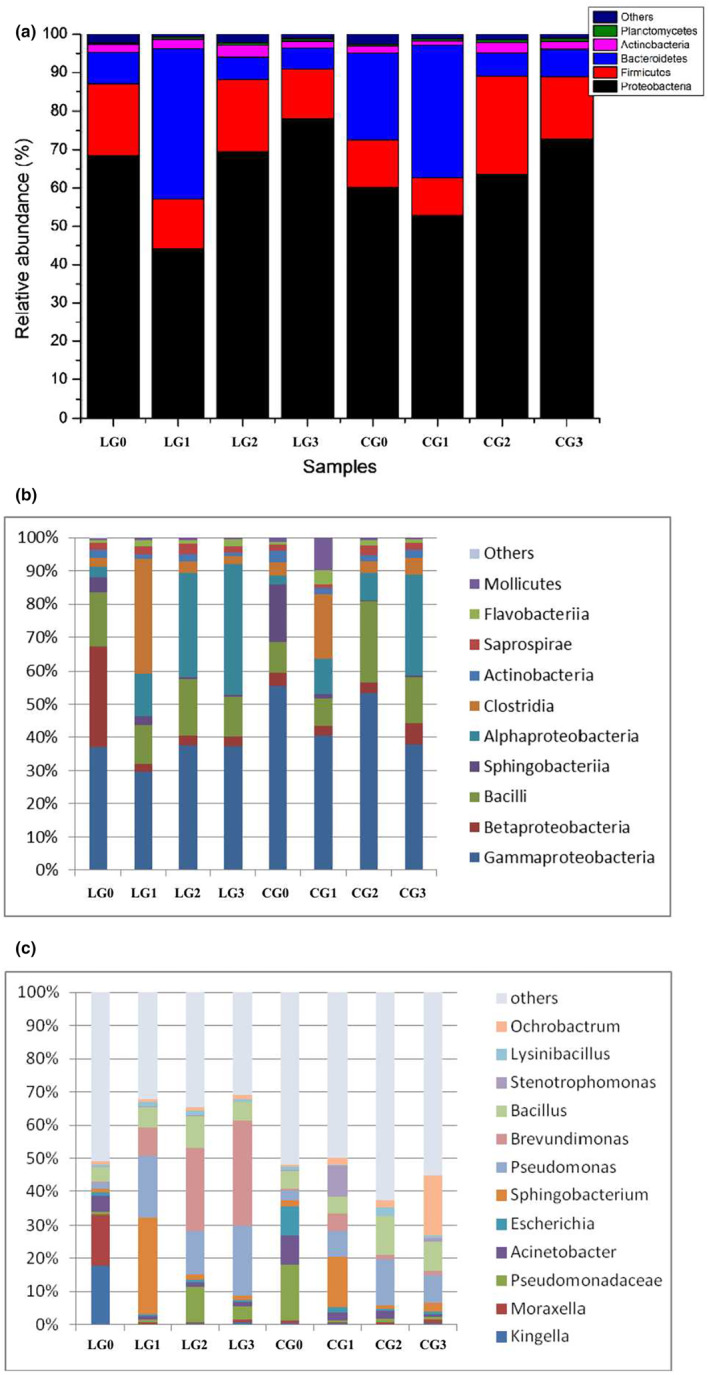
Relative abundance at the (a) phylum, (b) class, and (c) genus levels of dominant taxa of IQ‐degrading microorganisms in the digestive tract of camels.

The obtained sequences were compared with the SILVA database and the results showed that the most similar sequences were classified into 24 phyla, 61 classes, and 316 genera. As shown in Figure 3a, Proteobacteria, Actinobacteria, Bacteroidetes, Planctomycetes, and Firmicutes constituted the major phyla. Especially, the Proteobacteria dominated in the original microbial flora of the rumen (LG0) and intestine (CG0) samples. In the first‐generation samples (LG1 and CG1), the relative abundance of the Bacteroidetes increased, while by the second (LG2, CG2) and third (LG3, CG3) generation samples, the Proteobacteria re‐emerged as the dominant group.

The dominant bacteria in the original samples from the rumen (LG0) and intestine (CG0) of camels belonged to Gammaproteobacteria (Figure 3b). With IQ stress, the abundance of Sphingobacteriia in the samples of the first generation (LG1 and CG1) of rumen and intestine increased. The abundance of both Gammaproteobacteria and Alphaproteobacteria in the samples of the second (LG2) and third generation (LG3) of rumen increased and became the dominant group. The abundance of Gammaproteobacteria and Flavobacteriia in the second generation (CG2) of the intestinal tract increased and they became the dominant bacteria. Gammaproteobacteria and Alphaproteobacteria re‐emerged as the dominant flora in the third generation (CG3) of intestinal samples.

At the genus level (Figure 3c), the dominant genera in the original samples (LG0) of the rumen consisted of *Kingella* and *Moraxella*. *Sphingobacterium* and *Pseudomonas* were the dominant genera in their first‐generation samples (LG1). In rumen second‐ (LG2) and third‐generation (LG3) samples, *Brevundimonas* and *Pseudomonas* were dominant. The dominant genera in the original intestinal samples were *Pseudomonadaceae* and *Escherichia*. Under the stress of IQ, the dominant genera in the first‐generation samples (CG1) of the intestine changed, and *Sphingobacterium* and *Stenotrophomonas* became the dominant genera. In the second‐generation samples (CG2), the dominant genera were *Pseudomonas* and *Bacillus*, but by the third generation (CG3), the dominant genera were *Ochrobactrum*, *Pseudomonas*, and *Bacillus*.

### 3.4 Identification and phylogenetic analysis of IQ‐degrading bacteria

**FIGURE 4:**
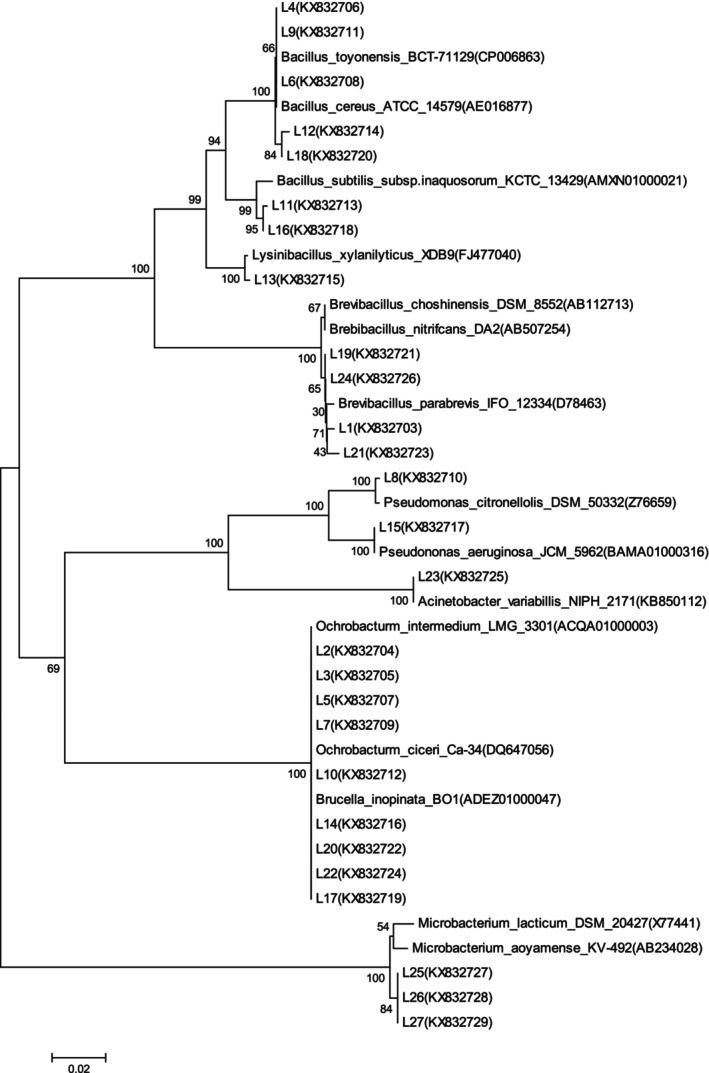
Phylogenetic tree of 27 IQ‐degrading strains in the rumen of camels.

**FIGURE 5:**
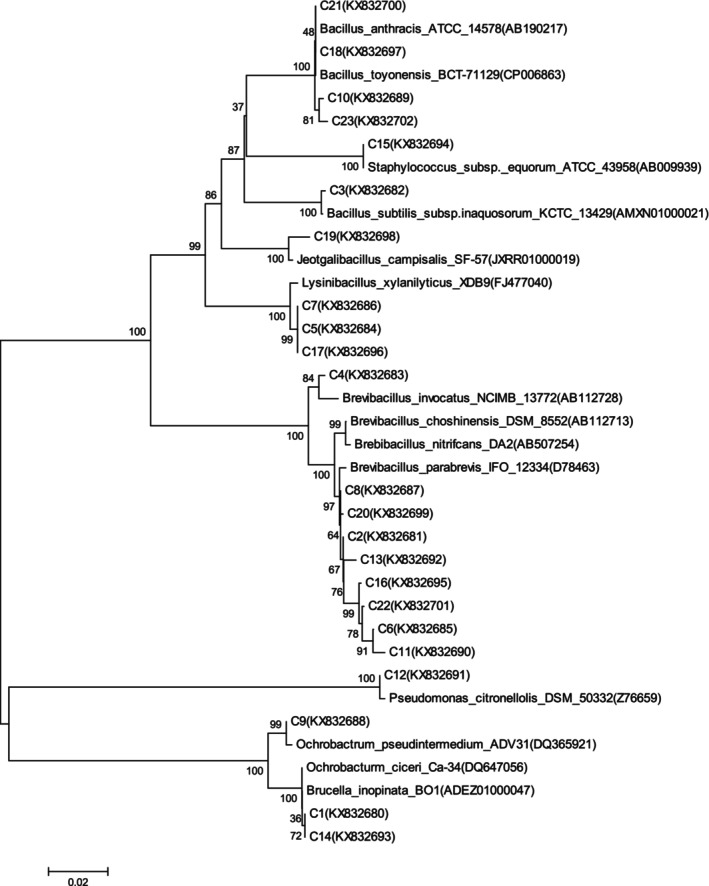
Phylogenetic tree of 23 IQ‐degrading strains in the intestine of camels.

The third generation of camel ruminal (LG3) and intestinal (CG3) contents was incubated with IQ as the only carbon source in solid isolation medium. Then, single colonies as degrading bacteria in the mixture were isolated and purified: Twenty‐seven strains from the rumen of camels, were numbered from L1 to L27, and 23 strains were from the intestine of camels, numbered as C1–C23. And these degrading bacteria were analyzed in GenBank (Tables 3 and 4), and the phylogenetic tree was constructed (Figures 4 and 5). The dominant genera were *Ochrobactrum*, *Bacillus*, and *Pseudomonas* of 27 strains from camel rumen (Table 3 and Figure 4). As for 23 strains from camel intestine (Table 4 and Figure 5), *Brevibacillus* and *Bacillus* were the dominant genera.

**TABLE 3:** Blast comparison results of 27 IQ‐degrading strains in the rumen of camel.

Strains	Accession number	Strains in GenBank database (accession no.)	Similarity (%)
L1	KX832703	*Brevibacillus parabrevis*; D78463	99
L2	KX832704	*Ochrobactrum intermedium* partial	99
L3	KX832705	*Ochrobactrum anthropi*	99
L4	KX832706	*Bacillus anthracis*; AB190217	100
L5	KX832707	*Ochrobactrum* sp. TSH8	99
L6	KX832708	*Ochrobactrum intermedium*. ACQA01000003	99
L7	KX832709	*Pseudomonas citronellolis*; Z76659	99
L8	KX832710	*Ochrobactrum intermedium* strain W18‐2	99
L9	KX832711	*Pseudomonas* sp. S‐47(2011)	99
L10	KX832712	*Bacillus anthracis*; AB190217	100
L11	KX832713	*Bacillus anthracis*. AB190217	99
L12	KX832714	*Ochrobactrum intermedium* strain NSB‐10	99
L13	KX832715	*Bacillus pumilus* strain X22	99
L14	KX832716	*Bacillus pumilus*. ABRX01000007	99
L15	KX832717	*Pseudomonas aeruginosa*. BAMA01000316	99
L16	KX832718	*Proteus mirabilis* strain BCr	99
L17	KX832719	*Bacillus anthracis*. AB190217	99
L18	KX832720	*Ochrobactrum* sp. TSH73	99
L19	KX832721	*Ochrobactrum intermedium*. ACQA01000003	99
L20	KX832722	*Brevibacillus parabrevis*. D78463	99
L21	KX832723	*Bacillus pumilus*. ABRX01000007	99
L22	KX832724	*Acinetobacter variabilis*. KB850112	99
L23	KX832725	*Brevibacillus parabrevis*; D78463	99
L24	KX832726	*Microbacterium lacticum*. *X*77441	99
L25	KX832727	*Microbacterium lacticum*. X77441	99
L26	KX832728	*Ochrobactrum* sp. SY 286	99
L27	KX832729	*Acinetobacter variabilis*. KB850112	99

**TABLE 4:** Blast comparison results of 23 IQ‐degrading strains in the intestine of camel.

Strains	Accession number	Strains in GenBank database (accession no.)	Similarity (%)
C1	KX832680	*Ochrobactrum* sp. TSH79	99
C2	KX832681	*Brevibacillus parabrevis*. D78463	99
C3	KX832682	*Bacillus subtilis* subsp. *inaquosorum*; AMXN01000021	99
C4	KX832683	*Brevibacillus invocatus*. AB112728	99
C5	KX832684	*Brevibacillus invocatus*. AB112718	99
C6	KX832685	*Brevibacillus parabrevis*. D78463	99
C7	KX832686	*Lysinibacillus xylanilyticus*. FJ477040	99
C8	KX832687	*Brevibacillus parabrevis*. D78463	99
C9	KX832688	*Enterobacter* sp. CZGRY7	99
C10	KX832689	*Bacillus anthracis*. AB190217	99
C11	KX832690	*Brevibacillus nitrificans*. AB507254	99
C12	KX832691	*Brevibacillus parabrevis*; D78463	99
C13	KX832692	*Brevibacillus parabrevis*; D78463	99
C14	KX832693	*Bacillus cereus* strain CP1	99
C15	KX832694	*Staphylococcus equorum* subsp. *equorum*; AB009939	99
C16	KX832695	*Bacillus* sp. wust‐c	99
C17	KX832696	*Lysinibacillus xylanilyticus*. FJ477040	99
C18	KX832697	*Bacillus anthracis*. AB190217	99
C19	KX832698	*Jeotgalibacillus campisalis*. JXRR01000019	99
C20	KX832699	*Brevibacillus parabrevis*. D78463	99
C21	KX832700	*Bacillus anthracis*; AB190217	100
C22	KX832701	*Brevibacillus parabrevis*. D78463	99
C23	KX832702	*Bacillus anthracis*; AB190217	99

### 3.5 Degrading capacity of bacteria in IQ

**FIGURE 6:**
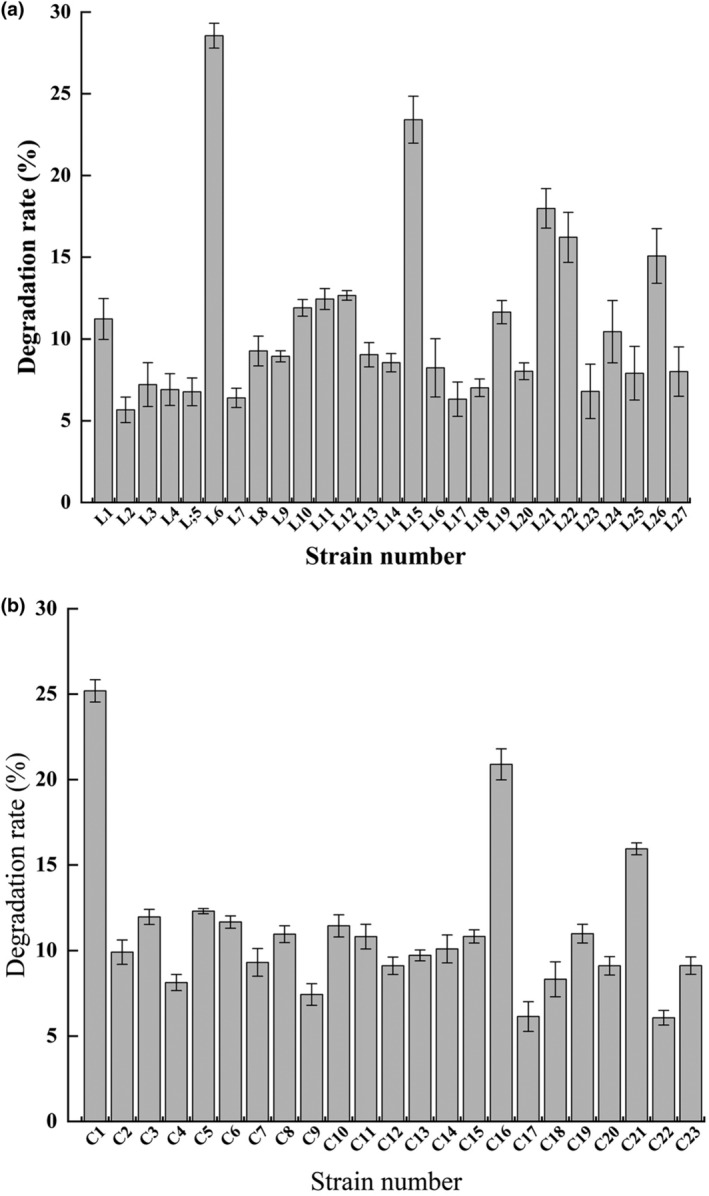
Degradation rate of IQ by (a) 27 strains of bacteria in the rumen and (b) 23 strains of bacteria in the intestinal samples of camels.

The microorganisms from the IQ‐stressed third‐generation cultures were screened to select degrading strains, and 50 strains were isolated and purified. Degrading ability of these 50 strains after 10 days of IQ‐stressed incubation is shown in Figure 6. The degrading capacity of IQ by strains from camel rumen was up to 28.55% and down to 5.38%, while the degrading capacities of strains from camel intestine varied from 5.98% to 25.19%. Two efficient degrading strains were screened from the rumen as L6 and L15, which were identified as *Ochrobactrum* and *Pseudomonas*, and two efficient degrading strains were screened from the intestine: C1 (*Ochrobactrum*) and C16 (*Bacillus*).

**FIGURE 7:**
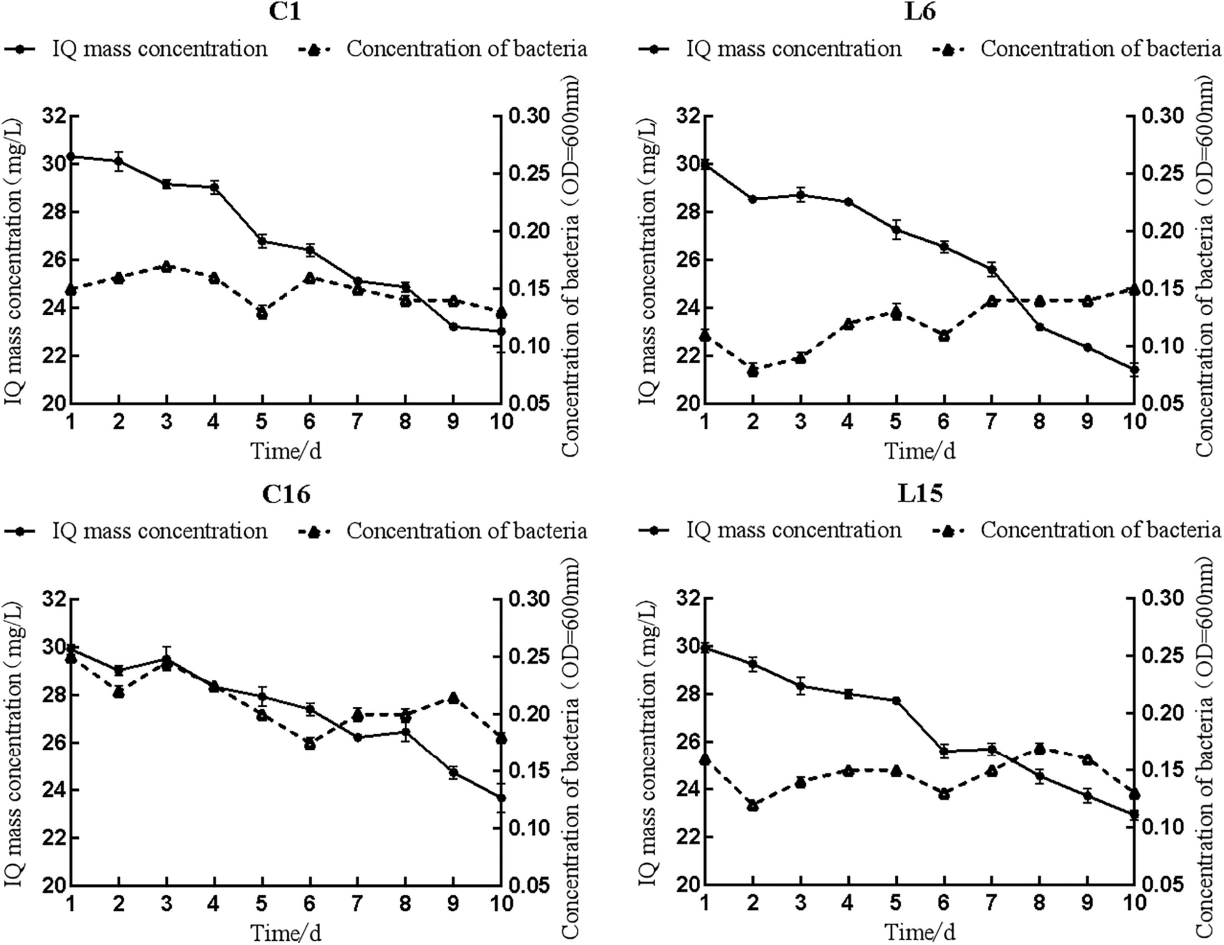
Growth and degradation curves of four highly effective degrading strains with IQ (30 mg/L) stressed.

During IQ‐stressed incubation, the growth curves and degradation curves of the four degrading strains are shown in Figure 7. Within 1–2 days, the strains were in a sluggish phase with a slow growth and a slight decrease in IQ concentration in order to adapt to the growth environment. And then, the rapid growth of the strains reached a stable phase with a high rate of utilization of IQ. From day 8 to day 10, the concentration of the four strains gradually decreased, and the concentration of IQ also decreased to the lowest. The degradation rates of C1, L6, C16, and L15 on IQ were 25.19%, 28.55%, 20.89%, and 23.41%, respectively.

### 3.6 Morphological changes of the bacterium during degradation

**FIGURE 8:**
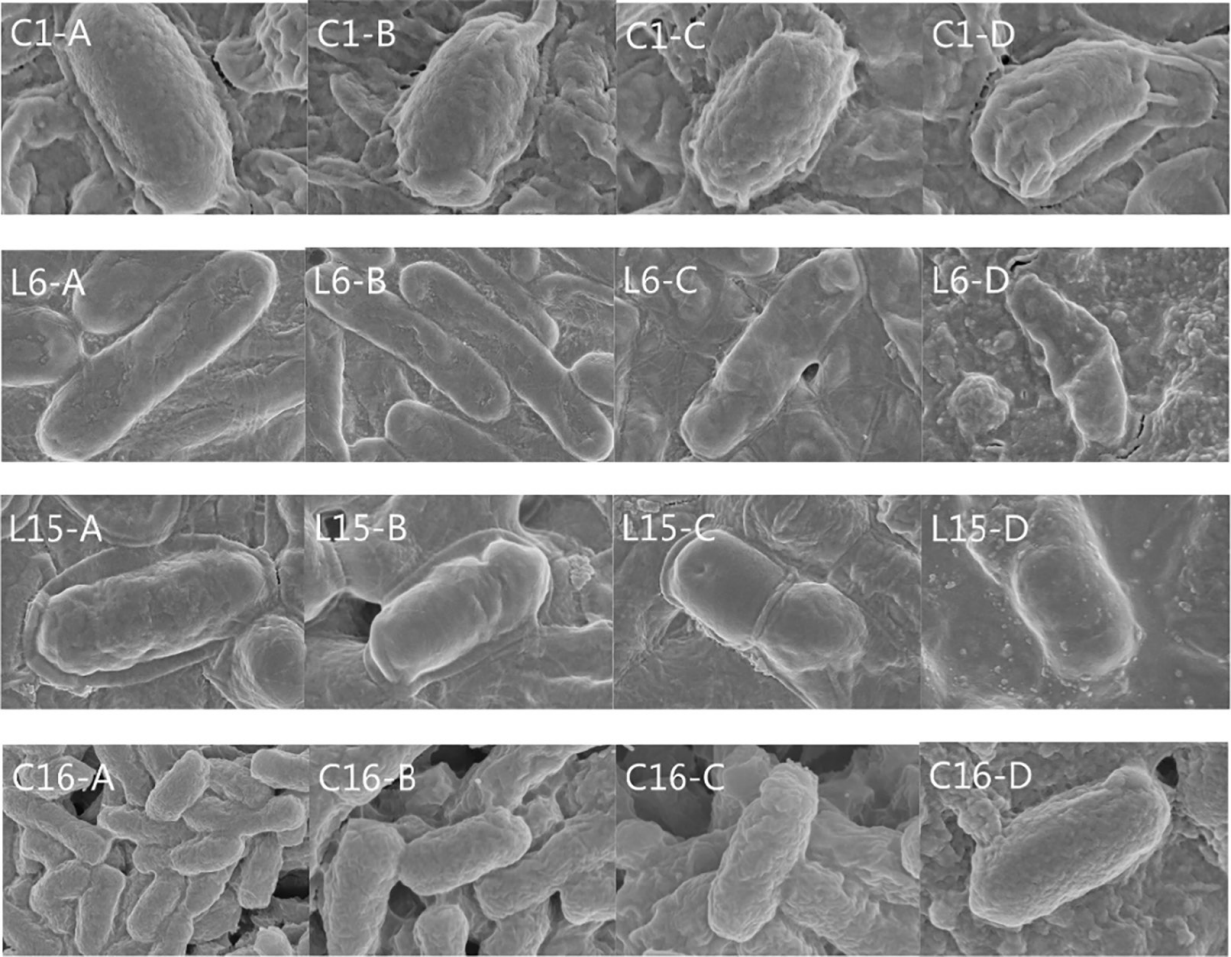
Morphological characteristics of four highly effective degrading strains with IQ (30 mg/L) stressed. A, B, C, and D represent samples of strains cultured on days 0, 3, 6, and 10, respectively.

Four degrading strains were screened by electron microscopy and the results are shown in Figure 8. After 3 days of stress incubation with IQ (30 mg/L), the C1, C16, L6, and L15 bacteria began to morphologically change. By day 6, there were obvious folds on the surface of the four strains. After 10 days of IQ‐stressed incubation, the cell surface folds of four strains deepened and became depressed, and C1 (*Ochrobactrum*), L6 (*Ochrobactrum*) and L15 (*Pseudomonas*) strains were severely deformed morphologically.

## 4 DISCUSSION

The current macrogenomic sequencing analysis of microorganisms in the camel's digestive tract revealed that Bacteroidetes, Firmicutes, and Proteobacteria were the dominant phyla in the rumen, with Bacteroidetes accounting for more than a half (Bhatt et al., 2013; Gharechahi et al., 2015). He et al. (2018) found that the dominant phyla in the rumen of camels were Firmicutes and Bacteroidetes, while the relative abundance of Firmicutes and Verrucomicrobia in the intestine was higher. Different growing environments and physiological conditions can lead to differences in the microbial composition of the rumen and intestine of camels. The first dominant bacteria in the rumen and intestine of camels were the Proteobacteria, the second dominant bacteria in the rumen were the Firmicutes, and the second dominant bacteria in the intestine were the Bacteroidetes.

Through three generations of IQ stress culture, rumen and intestinal microorganisms adapted to the growth environment where IQ was the only source of carbon, resulting in a gradual dominance of IQ‐dependent flora, thus changing the original microbial composition of the rumen and intestine. As the number of culture generations increased, the relative abundance of the Proteobacteria in the rumen and intestine increased dramatically, accounting for more than 70% of the total, and it was possible that the Proteobacteria in the camel's digestive tract responded positively to the stress of IQ.

Microorganisms were further isolated and purified from IQ‐stressed third‐generation cultures. Four strains were finally screened for good IQ clearance, including two strains of *Ochrobactrum*, one strain of *Pseudomonas*, and another strain of *Bacillus*. Results were accordant with the observation of relative abundance analysis. The strains of third generation of the rumen contents were mainly attributed to *Brevundimonas* and *Pseudomonas* and strains in intestine were mainly as *Ochrobactrum*, *Bacillus*, and *Pseudomonas*. Results of bacteria identification and their phylogenetic tree analysis also showed greater abundance of *Ochrobactrum*, *Bacillus*, *Pseudomonas*, and *Brevundimonas* genera, which indicated bacteria of these dominant genera exerted stronger tolerance to IQ.

Microorganisms of the genus *Ochrobactrum* could be found in fermented foods, which might result from their strong resistance to heat, drought, and high‐salt conditions (Chen et al., 2021; Hu et al., 2020; Hui et al., 2017). These strains were also able to degrade organic pollutants such as aromatic compounds, pesticides, and veterinary drugs, thus their application in food safety and bioenvironmental remediation has an important potential (Ma et al., 2022; Wozniak‐karczewska et al., 2018).

*Bacillus* spp. have a strong antibacterial activity and good degradation of many pesticides, such as pentachloronitrobenzene (PCNB), fluazinam, and clofenotane (DDT) in food (Wozniak‐karczewska et al., 2018). López et al. (2021) found that *Bacillus* spp. derived from the intestinal microflora were able to tolerate or degrade the endocrine disruptor bisphenol A (BPA). Studies have confirmed that *Bacillus* spp. can express enzymes catalyzing the cleavage of the C‐C bonds in aromatic rings and thus degrade organic pollutants. After treatment with polycyclic aromatic hydrocarbons (PAHs), certain proteins in *Bacillus* spp. were involved in various biological processes such as energy metabolism, biosynthesis, transmembrane transport, and oxidative stress (Zhu et al., 2019). *Bacillus* spp. may be an effective strain for degrading foodborne toxins like PAHs, HAs, and pesticides. *Pseudomonas* was also capable of degrading HAs (Zhao et al., 2012). The genera *Bacillus* and *Pseudomonas* were found in fermented foods, especially the *Bacillus* spp. were the starter strains for soybeans, cereals, or fishes to produce tofu, natto, or fish sauce (Divyashree et al., 2024; Li et al., 2023; Obinze et al., 2022; Sivamaruthi et al., 2022). Results indicated the strains of *Ochrobactrum*, *Bacillus*, and *Pseudomonas* from the digestive tract of camels provided a microbial resource for food safety to biodegrade various toxins.

Four efficient degrading strains were finally screened from the camel rumen and intestine: L6 (*Ochrobactrum*), L15 (*Pseudomonas*), C1 (*Ochrobactrum*), and C16 (*Bacillus*). Their degrading ability ranging from 20% to 30% was correspondingly high (Stidl et al., 2008; Vanhaecke et al., 2008). Multiple uses of these bacteria may increase the IQ‐degrading capacity, because these bacteria showed different biodegrading pathways, which may contribute to synergy of IQ decreasing (Nogacka et al., 2019).

Microbes may exert biodegrading ability by binding IQ or other toxins and destructing their chemical structures (Nogacka et al., 2019; Shao et al., 2022). Microbial cells, cell debris, and cell wall skeleton could bind IQ or other toxic substances. Some enzymes were released from microbes to catalyze the destruction of these toxic substances (Farag et al., 2022; Sallan et al., 2023). Microbes, in turn, were damaged by these substances. During the IQ‐stressed incubation, deformation and lysis were observed on the surface of the bacteria by electron microscopy, indicating that IQ has damaged the growth of the degrading strain. The morphological damage to the four strains may be caused by the poor nutritional conditions of the inorganic salt medium, which used IQ as the only carbon source. Detailed biodegrading mechanism needs to be further revealed.

Microorganisms that could degrade HAs are mainly from polluted environments such as soil and water bodies (Wang et al., 2015; Zhao et al., 2012). This experiment was the first to obtain bacteria from the digestive tract of camels that could degrade IQ, revealing the degrading characteristics of bacteria. Therefore, this research was valuable for studying the degradation of food‐borne toxic and harmful substances. Based on the study, the application of microbial resources in the animal digestive tract will be expanded to provide data for microbial degradation of foodborne pollutants.

## AUTHOR CONTRIBUTIONS

**Jialing Lin:** Software, Data curation. **Chuanhui Zeng:** Investigation, Methodology. **Xueli Li**: Writing – Review and Editing, Project administration. **Qin Tang**: Methodology, Writing – original draft. **Jing Liao**: Resources and Project administration. **Yan Jiang**: Conceptualization, Project administration. **Xianchun Zeng**: Conceptualization, Visualization, Resources, Funding acquisition.

## CONFLICT OF INTEREST STATEMENT

The authors declare that they have no conflict of interest.

## ETHICS STATEMENT

Ethics approval was not required for this research.

## Data Availability

The data that support the findings of this study are available from the two corresponding authors upon reasonable request.
